# Asymmetrical bilateral sternoclavicular joint dislocation combined with bilateral clavicular fracture: A case report

**DOI:** 10.1097/MD.0000000000016359

**Published:** 2019-07-12

**Authors:** Haifeng Wang, Chongyang Wang, Jianwei Ruan, Weiqian Wu

**Affiliations:** a Department of Orthopedics; b Department of Respiration, Taizhou Municipal Hospital, Taizhou, Zhejiang, China.

**Keywords:** bilateral, clavicular fracture, dislocation, sternoclavicular joint

## Abstract

**Rationale:**

Asymmetrical bilateral sternoclavicular joint (SCJ) dislocation consists of posterior SCJ dislocation on one side and anterior SCJ dislocation on the other side. This is an extremely rare injury and only a few cases have been reported in the literature. If not been diagnosed timely and accurately, asymmetrical bilateral SCJ dislocation can be life-threatening.

**Patients concerns:**

We experienced a patient who has a life-threatening posterior dislocation of right SCJ and anterior dislocation on the left SCJ combined with bilateral clavicular fracture after a traffic accident.

**Diagnoses:**

A computed tomography (CT) scan with three-dimensional reconstructions of SCJ showed potentially life-threatening posterior dislocation of right SCJ and anterior dislocation on the left SCJ combined with bilateral clavicular fracture.

**Interventions:**

Because of failed attempts at closed reduction, electively surgical intervention was made. We repaired the ruptured joint capsule and ligaments and fixed bilateral SCJ by Kirschner wire during the operation.

**Outcomes:**

Three-dimensional CT scans confirmed bilateral SCJ reduction and alignment after operation 1 week as well as at the 2-month follow-up.

**Lessons:**

SCJ dislocation is an extremely rare and life-threatening injury. The aim of the operation is to repair the ruptured joint capsule and its ligaments and to fix the dislocated joints.

## 1 Introduction

Because of the protection of strong ligaments, it often requires greater forces to cause sternoclavicular joint (SCJ) dislocation. Compared with all injuries of the shoulder girdle, SCJ dislocation is a rare injury and can be classified into 2 types namely posterior and anterior dislocation. Anterior SCJ dislocation accounts for about 3% of all injuries to the shoulder girdle^[1–3]^ and represents 1% of all joint dislocation.^[2]^ The incidence of anterior SCJ dislocation is approximately 9 to 20 times greater than that of posterior dislocation.^[4–6]^ As 95% are unilateral, bilateral SCJ dislocation is more rare.^[7]^

Because posterior SCJ dislocation is a very rare injury and there was no obvious findings on physical examination. Therefore, the orthopedist may not be aware of this injury promptly. Besides, there are some important thoracic structures behinding the SCJ, such as great vessels, nerves, esophagus, trachea. Therefore, if not been diagnosed timely and accurately, posterior SCJ dislocation can be life-threatening.

The diagnosis of SCJ dislocation relies on a careful physical examination and evaluation. In suspected SCJ dislocations, computed tomography (CT) scan with a three-dimensional reconstruction has become the imaging study of choice.^[1,6,8,9]^ Open reduction and internal fixation may be considered if closed reduction is unsuccessful or recurrent dislocation.

We experienced a patient who has a potentially life-threatening posterior dislocation of right SCJ and anterior dislocation on the left SCJ combined with bilateral clavicular fracture and this case acquired a satisfactory result. After reviewing the literature, we found there were no similar cases reported in the world.

## 2 Case report

We acquired the informed written consent for publication of this case report and accompanying images from the patient. Because this is a case report, ethics committee approval was waived.

A 28-year-old woman was driving a motorcycle alongside the truck. The truck swerved to the right suddenly and ran over her. She lost consciousness when paramedics arrived on the scene and was rapidly sent to the emergency department. Multiple abrasions to the chest and abdomen were seen and there was no obvious deformity on both side of SCJ.

**Figure 1:**
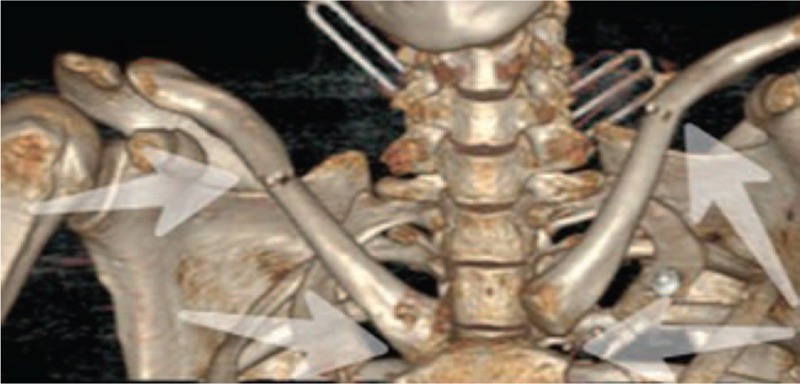
A computed tomography (CT) scan with three-dimensional reconstructions of sternoclavicular joint (SCJ) showed potentially life-threatening posterior dislocation of right SCJ and anterior dislocation on the left SCJ combined with bilateral clavicular fracture. CT = computed tomography, SCJ = sternoclavicular joint.

A full-body CT scan was done for further treatment after establishing respiratory and systemic circulation, which revealed comminuted fracture of pelvis, hemopneumothorax, multiple rib fractures, bilateral lung contusion. A CT scan with three-dimensional reconstructions of SCJ showed potentially life-threatening posterior dislocation of right SCJ and anterior dislocation on the left SCJ combined with bilateral clavicular fracture (Fig. 1). She was brought to the intensive care unit (ICU) and discharged in stable condition a few dates later.

**Figure 2:**
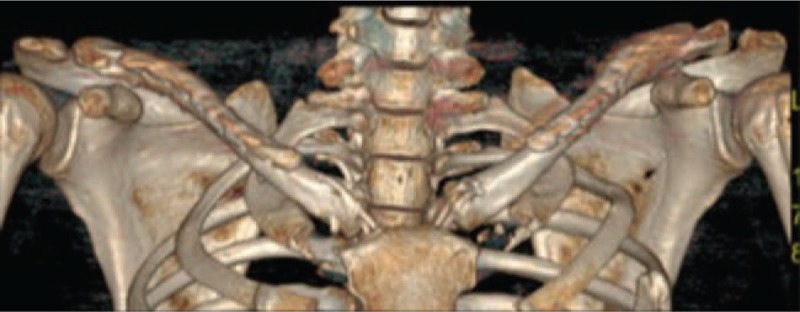
Three-dimensional computed tomography (CT) scans confirmed bilateral sternoclavicular joint (SCJ) reduction and alignment after operation 1 week. CT = computed tomography, SCJ = sternoclavicular joint.

Because of failed attempts at closed reduction, electively surgical intervention was made. We repaired the ruptured joint capsule and ligaments and fixed bilateral SCJ by Kirschner wire during the operation. Three-dimensional CT scans confirmed bilateral SCJ reduction and alignment after operation 1 week (Fig. 2) as well as at the 2-month follow-up.

## 3 Discussion

There are many joints and bones surrounding shoulder girdle, which receive more energy from blunt trauma. Meantime the SCJ's stability derived from well reinforced ligamentous structures (costoclavicular ligament, interclavicular ligament, posterior and anterior sternoclavicular ligaments, capsular) surrounding the joint.^[10]^ Therefore, compared with all injuries of the shoulder girdle, traumatic SCJ dislocation is a rare injury. A outcome of study by Spencer et al^[11]^ indicated that the strongest ligamentous stabiliser is posterior capsule. In terms of direction of dislocation, the incidence of anterior SCJ dislocation is approximately 9 to 20 times greater than that of posterior dislocation.^[4–6]^ As 95% are unilateral, bilateral SCJ dislocation (nontraumatic or traumatic) is more rare.^[7,9,12,13]^ In general, it requires a powerful force of vector to cause sternoclavicular dislocation, such as falls from height, and motor vehicle accidents. Asymmetrical SCJ dislocations consist of posterior SCJ dislocation on one side and anterior SCJ dislocation on the other side. This situation means that powerful forces of vector to cause SCJ dislocation come from anterior and posterior directions, respectively. This is an extremely rare injury and only a few cases have been reported in the literature.^[3,13]^ We experienced a patient who has a potentially life-threatening posterior dislocation of right SCJ and anterior dislocation on the left SCJ combined with bilateral clavicular fracture. To our knowledge, there were no similar cases reported in the world to date.

There was often no obvious findings on physical examination for patients in a coma with posterior SCJ dislocation which is an extremely rare injury. The orthopedist may not be aware of this injury promptly. But here are some important thoracic structures behind the SCJ, such as great vessels, nerves, esophagus, trachea. If not been diagnosed timely and accurately, posterior SCJ dislocation can be life-threatening.

The diagnosis of SCJ dislocation relies on a careful physical examination and evaluation. In suspected SCJ dislocations, CT scan with a three-dimensional reconstruction has become the first choice of imaging examination.^[1,6,8,9]^

Closed reduction under general anesthetic or with sedation should be attempted in the operating room for patients with acute anterior and posterior SCJ dislocation (within 7–10 d). However, some life-threatening complications during the closed reduction of posterior SCJ dislocation have been reported in the literature,^[4,14–16]^ such as neurovascular, tracheal, esophageal, and other injury. Hence, we need to pay more careful evaluation for reduction of posterior SCJ dislocation. After closed reduction for anterior SCJ dislocation, rates of redislocation have been reported in the literature from 21% to 100%.^[4,17,18]^ We should inform patients there is a high risk of persistent SCJ instability after closed reduction of anterior dislocation and this instability has minimal impact on functionality and be managed with non-surgical therapy in the vast majority.

If closed reduction is unsuccessful, recurrent dislocation or persistent symptomatic SCJ instability, open reduction should be adopted. To date there is no uniform operative procedure. The aim of the operation is to repair the ruptured joint capsule and its ligaments and to fix the dislocated joints using wire suture, internal fixation, allograft or autograft tendon, myotendonous transfer, fascia lata, and so on.^[19–25]^ Excision arthroplasty of the medial clavicle is an alternative procedure when good joint capsule and ligament cannot be reconstructed, but there was a poor postoperative outcomes.^[26]^

In our case report, we used Kirschner wires to fix bilateral SCJ dislocation and anatomical plates to fix bilateral fracture of clavicle. During the 2-month follow-up after the operation, the patient did not report discomfort symptoms and acquired good shoulder motion. To our knowledge, this is the first case of a potentially life-threatening posterior dislocation of right SCJ and anterior dislocation on the left SCJ combined with bilateral clavicular fracture.

## Author contributions

**Writing – original draft:** Hai feng Wang, Jian wei Ruan.

**Writing – review & editing:** Wei qian Wu, Chong yang Wang.
